# A Review of Web Portal Use by Oncology Patients

**DOI:** 10.29245/2578-2967/2018/6.1154

**Published:** 2018

**Authors:** Steven S. Coughlin, Lee Caplan, Lufei Young

**Affiliations:** 1 Department of Population Health Sciences, Medical College of Georgia, Augusta University, Augusta, GA; 2 Research Service, Charlie Norwood Veterans Affairs Medical Center, Augusta, GA; 3 Morehouse College of Medicine, Department of Community Health and Preventive Medicine, Atlanta, GA; 4 College of Nursing, Augusta University, Augusta, GA

**Keywords:** Cancer, Self-Management, Electronic Health Record, Health Information Technology, Patient Web Portals

## Abstract

**Background:**

Patient portals and other Internet-based technologies have been increasingly used to improve cancer care coordination. Patient portals may introduce special considerations in oncology populations where longitudinal outpatient care is often more intensive than in most other specialties.

**Methods:**

This article, which is based upon bibliographic searches in PubMed, reviews the literature on web portal use by cancer patients. Articles published in English from 2000 to August 2018 were identified using the following MeSH search terms and Boolean algebra commands: web portal AND cancer. Information obtained from bibliographic searches (title and topic of article, information in abstract, and keywords) was used to determine whether to retain each article identified in this way.

**Results:**

A total of 263 article citations were identified in the bibliographic searches. Of these, 10 met the eligibility criteria. A variety of study designs were used including focus groups, usability testing, in-person interviews, questionnaire surveys, retrospective cohort, and non-randomized trial. Cancer patients had reached modest levels of portal use. Increased portal use has been associated with younger age, white race, and higher socioeconomic status. Most cancer patients used portals to look up testing results and provide notes, but had difficulty in interpreting the results appropriately.

**Conclusions:**

Our study adds to the growing evidence that patient portals play a significant role in promoting self-management in cancer survivors. Additional studies are needed to determine factors influencing portal use, so effective interventions can be developed to enhance portal use.

## Introduction

Cancerand its treatment result in a wide range of self-management challenges that require effective care coordination, including patient-provider communication, monitoring of adverse events, and appropriate patient follow-up^1,2^. Effective self-management requires cancer patients taking an active role in their care and being kept well informed about their treatment plan and options^3^). Patient portals and other Internet-based technologies have been increasingly used to improve cancer care coordination1.

Although the use of patient portals has been evaluated in primary care populations with non-cancerous conditions^4^, there has only recently been interest in evaluating patient portals in patients with cancer and cancer survivors. Patient portals may introduce special considerations in oncology populations where on-going outpatient care is often more intensive than in most other chronic disease management5. Patient portals allow patients to have access to their clinical results that provide important information on disease progression and treatment outcomes. However, patients with low cognitive functioning and/or low health literacy skills could have difficulty in understanding these clinical results, resulting in heightened anxiety and confusion when the appropriate medical interpretation is not available ^5^. In addition, cancer patients often suffer from a range of symptoms due to disease progression, treatment and associated comorbid diseases. Patient-reported symptoms are significantly associated with medication adherence, health-related quality of life (HRQoL), hospitalization and mortality. Despite their importance, symptoms are often unrecognized and undertreated by health providers because of delayed or absence of patient -provider communication. Secure messaging via the patient portal can provide an effective platform for ongoing patient-provider communication and streamline the interaction, leading to better symptom management ^5,6^. On the other hand, providers may also be concerned about increased workload due to more frequent communication with patients via secure messaging^7^.

The increased interest among providers and researchers in web portal use in oncology populations follows efforts at health care reform and continued advances in information technologies. Stakeholders view patient portals, and parallel advances in eHealth such as personal health records and electronic medical records, as an opportunity to leverage information technology to support patient self- management and improve patient-provider communication between office visits ^4,8^ introduction of web-based, patient - centered health. careThe information systems linked to a patient’s electronic medical record (patient web portals) constitute an important development in oncology care.

## Materials and methods

Thepresent review is based on bibliographic searchers in PubMed and relevant search terms. Articles published in English from 2000 to August 2018 were identified using the following MeSH search terms and Boolean algebra commands: web portal AND cancer. The searches were not limited to words appearing in the title of an article. The searches were not limited to studies in a particular country or geographic region of the world. Information obtained from bibliographic searches (title and topic of article, information in abstract, and keywords) was used to determine whether to retain each article identified in this way. One of us (S.S.C.) reviewed the results of the bibliographic searches to determine whether each article was eligible for inclusion.

## Results

Atotal of 263 article citations were identified in the bibliographic searches. Of these, 10 met the eligibility criteria. A variety of study designs were used including focus groups, usability testing, in-person interviews, questionnaire surveys, retrospective cohort, and non-randomized trial.

Pai et al.^3^ provided 22 prostate cancer patients with access to a web-based personal health record (PHR) and then surveyed them at the end of the study period. Of the 17 patients who completed the study, 29% encountered minor difficulties with the (PHR). The two most commonly accessed medical records were laboratory test results and transcribed doctor’s notes. Ninety four percent were satisfied with the access to their medical records. 65% felt that the PHR helped them to communicate better with their physicians, 83% found new and useful information that they would not have received by talking to their health care providers, and 88% said that they would continue to use the PHR.

In a retrospective cohort study of 6,495 patients at a cancer center who enrolled in a web portal, Gerber et al^5^ found that, from 2007 to 2012, the median number of portal log-ins was 57 per patient. The most common portal actions were viewing test results (37%), viewing and responding to clinic messages (29%, and sending medical advice requests (6.4%). Increased portal use was significantly associated with younger age, white race, and an upper aerodigestive cancer diagnosis. Over the study period, the average number of patient log-ins per year more than doubled.

Girault et al.^1^ surveyed 1,371 outpatients at a comprehensive cancer center about their use of Internet-based technologies (patient portals, websites and applications) and attitudes towards such technologies. Age and socioeconomic status were negatively associated with the use of internet-based technologies (<0.001). Regarding patients’ expected benefits, a wide majority valued its use in health care, especially as a way to enhance communication with providers.

Kuijpers et al.^9^ conducted in-person interviews of 16 cancer survivors to evaluate content and graphic design of a prototype interactive web portal for breast and lung cancer survivors. Usability testing of the portal was completed with the assistance of 7 cancer survivors. Based on the initial version draft, survivors selected the preferred graphic design, approved the features and provided suggestions for the content. Usability testing revealed that it was relatively easy to navigate the website and use the different features.

To obtain input about possible features of an interactive web portal, Kuijpers et al.^10^ conducted five focus groups with 35 breast and lung cancer survivors and four focus groups with 31 health professionals. Important themes included fulfillment of information needs, communication, motivation, quality of feedback, and supervision. Cancer survivors were primarily interested in features that could fulfill their information needs, i.e., survivorship care plan,access to their electronic medical record, and an overview of appointments. Health professionals considered patient reported outcomes and telemonitoring as the most useful features.

Kuijpers et al.^11^ conducted a four- month trial of an interactive web portal involving 92 breast cancer survivors.Overview of appointments and access to the electronic medical record were most frequently used features and most highly valued. Average website user satisfaction was 3.8 on a 5-point scale. Patient activation scores did not change significantly. Three domains of the SF-36 measure of HRQoL (role functioning – emotional, mental health, and social functioning) and median vigorous physical activity improved significantly over time.

Laccetti et al.^7^ conducted a retrospective cohort study of 289 cancer center providers and clinic staff who performed patient portal activities. From 2009 to 2014, 289 employees performed 740,613 patient web portal actions and received 117,799 messages. Seventy-seven percent of actions were performed by nurses, 11% by ancillary staff, 6% by midlevel providers, and 5% by physicians. On average, 6.3 staff web portal actions were performed per patient-initiated message

To evaluate a patient web portal, Groen et al. ^2^ surveyed 37 lung cancer patients, conducted a focus group, and analyzed interactive patient portal log data. The majority of responses (82%) about using the interactive patient portal were positive; 69% saw it as a valuable addition happy with the web portal and other internet-based technology. There were some common themes that came out of some of the studies, including better communication between patients and their providers and improved patient access to their electronic medical records. to care, and 56% perceived increased control over their health. However, no significant changes were observed

To obtain a better understanding of communicative behaviors and perceptions of a patient web portal and how it is utilized in oncology, Alpert et al. ^12^ conducted in-depth, semi-structured interviews of 35 cancer patients and 13 oncologists. Content analysis suggested that portals help to enhance participation during in-person consultations, increase patients’ self-advocacy, and build rapport with providers. Patients’ level of comfort with reviewing information via the portal depended upon the severity of the test. Oncologists worried about patient anxiety and widening health disparities but noted that the portal can motivate them to expedite communication about test results.

Schultz and Alderfer^13^ conducted one-on-one semistructured interviews of 19 caregivers of children with cancer. Caregivers recognized advantages of portal use including getting results “fast,” being able to visualize trends in results, “keeping a record,” and not interfering with clinic flow. Perceived disadvantages included the results being “complicated” or easily misunderstood, and learning results prior to disclosure by care team.

## Discussion

This review showed patient portals were underutilized among cancer patients. Similar to other’s findings^14^, there were significant differences in patient characteristics between users and nonusers. Increased portal use has been associated with younger age, white race, and higher socioeconomic status (Gerber et al. 2014; Girault et al.2015). Overall, both cancer patients and providers seemed happy with the web portal and other internet-based technology. There were some common themes that came out of some of the studies, including better communication between patients and their providers and improved patient access to their electronic medical records.

Despite the obvious benefits of online portal use, two of the studies found disadvantages of the internet-based technology, and these included patient anxiety, patients not understanding their results, and patients learning results prior to disclosure by their care teams^12, 13^. All of these disadvantages revolve around patient anxiety, as patients not understanding their results and learning them prior to disclosure by their care teams can both lead to patient anxiety by leaving the patients in limbo for a certain length of time.

Twiddy^15^ described six barriers to the use of online portals. These barriers included physicians who were not convinced of the value of portals or had questions and concerns about the technology; physician finances as portal messaging is generally not reimbursed, and the use of the portal could reduce the need for the patient to see the physician and pay for a visit; the practice and medical staff as portal messaging could force the staff to answer more online messages and to change their routine for doing things; patient resistance to changing the way in which they communicate with their physicians; security and privacy concerns; and patient limitations such as age, income, or language preventing them from using a portal. In the present review, increased portal use was associated with younger age, white race, and higher socioeconomic status (Gerber et al. 2014; Girault et al. 2015). In addition, portals were found to increase patient participation during in-person oncology consultations (Alpert 2018).

Adler^16^ commented on the article by Twiddy. He observed that patients say that they want online access to both their physicians and their medical records, but the problem is that over three-fourths of them don’t enroll. He asked patients why they don’t enroll. Some are concerned over privacy, others have trouble using the computer and would just prefer to call and make appointments and leave messages, and others just don’t see the benefits.

Tarver et al^17^ explored trends over time in the use of online patient-provider communication tools using the Health Information National Trends Survey (HINTS) as a follow-up to a study by Beckjord, et al.^18^. This earlier study had found a low prevalence of online patient-provider communication, but which statistically significantly increased from 7% of internet users in 2003 to 10% in 2005. Tarver et al.^17^ found the prevalence to be 14% in 2008, 19% in 2011, and 30% in 2013. They also found that the proportion of internet users communicating online with their health care providers significantly increased between 2003 and 2013 with the odds increasing from 1.31 to 5.77.

In the earlier study, internet users who had more years of education, lived in a metropolitan area, reported poorer health status, or had a personal history of cancer were more likely to have used online patient-provider communication, while in the latter study, age, having health insurance, having a history of cancer, and living in an urban area were associated with internet users communicating online with providers. Shenson, et al.^19^ explored the use of secure messaging in a patient portal by surgeons. They found that the proportion of outpatient interactions conducted through secure messaging increased significantly from 5.4% in 2008 to 15.3% in 2010 (p < 0.001) with all surgical specialties experiencing growth.

Proponents of online portals argue that communication between patients and their care teams can be automatically appended to their electronic health records, which would prevent messages from being inaccurately given over from person to person before reaching the doctor. In addition, portal messaging can overcome the patients forgetting what the doctor told them in the office or over the telephone by providing a permanent written communication to which the patients can refer to. Also, the volume of patients might actually increase as patients who benefited from the portal might be more willing to make an appointment to see the physician, and as a result the physician’s revenue will not decrease.

Several limitations of this review originated from the original studies. First, there is a lack of clear evidence regarding the effectiveness of a patient portal as a tool in reducing adverse events. Hence, future investigation is needed to examine the impact of patient portal use on adverse events when engaging patients as safety partners. Second, the subsequent healthcare utilizations following the portal use were not examined in the selected studies. It is crucial to know whether portal use can improve health service use efficiency, reduce office visits, emergency department visits, and hospitalizations. The assumption was that if patients could view personal health information, they will be more informed, capable of managing their own health with less unplanned, and episodic healthcare utilizations (e.g., emergency department use or re-hospitalizations). This expectation has not been validated in our review. Third, all the selected studies failed to report the interaction between self-management knowledge and health literacy on portal use in cancer patients. Our review viewed the great challenges encountered by cancer patients in understanding testing results, but did not examine the causes of the challenges. Whether the barrier with understand testing results is associated with self-management knowledge and health literacy is unknown. A cancer diagnosis is a stressful life event, therefore, cancer patients’ information-seeking behavior was more prominent than other patients, which becomes a coping strategy to overcome uncertainties. The selected studies did not report the relationship between information-seeking behavior and portal use. A further limitation of this review is that the use of search terms other than “patient web portal” and “cancer” could have resulted in more studies being identified. However, we reviewed the references of review articles on patient web portals.

## Conclusion

Thereview showed that cancer patients had reached modest levels of portal use. Portal use is associated with several sociodemographic factors. Most cancer patients used portals to look up testing results and provide notes, but had difficulty in interpreting the results appropriately. Our study adds to the growing evidence that patient portals play a significant role in promoting self-management in cancer survivors. Additional studies are needed to determine factors influencing portal use, so effective interventions can be developed to enhance portal use.

## Figures and Tables

**Table 1.** Studies of web portal use by cancer patients.

Study	Sample	Design	Outcomes	Results
Pai et al. 2013	22 prostate cancer patients who received access to a web-based personal health record (PHR)	End-of-study survey	Usability, satisfaction, and concerns with provider	Of the 17 patients who completed the study, 29% encountered minor difficulties with the (PHR). The two most commonly accessed med- ical records were laboratory test results and transcribed doctor’s notes. 94% were satisfied with the access to their medical records, 65%
Gerber et al. 2014	6,495 patients at a cancer center who en- rolled in a web portal	Retrospective cohort	Increased portal use and portal functions used	From 2007 to 2012, the median number of portal log-ins was 57. The most common portal actions were viewing test results (37%), viewing and responding to clinic messages (29%0, and sending medical advice requests (6.4%). Increased portal use was significantly associated with younger age, white race, and an upper aerodigestive cancer diagnosis. Over the study period, the average number of pa- tient log-ins per year more than doubled.
Girault et al. 2015	1,371 outpatients (median age 53.4 years) at a compre- hen-sive cancer center	Questionnaire-based survey	Internet-based technologies (patient portals, websites and applications) usage and attitudes	Age and socioeconomic status were negative- ly associated with the use of internet-based technologies (<0.001). Regarding patients’ ex- pected benefits, a wide majority valued its use in health care, especially as a way to enhance communication with providers.
Kuijpers et al. 2015	23 cancer survivors	In-person interviews to evaluate content and graphic design of an interactive web portal for breast and lung cancer survivors, and usability testing of a prototype among 7 cancer survivors.	Content analysis was used to analyze the data of both the in- terviews and usability tests	Based on the first draft, survivors selected the preferred graphic design, approved the features and provided suggestions for the content. Usability testing revealed that it was relatively easy to navigate the website and use the different features.
Kuijpers et al. 2015	21 breast cancer sur- vivors, 14 lung cancer survivors, and 31 health profession-als	Five focus groups with cancer survivors and four focus groups with health professionals, to obtain input about possible features of an interactive web portal	Data were analyzed using content analysis	Important themes included fulfillment of in- formation needs, communication, motivation, quality of feedback, and supervision. Cancer survivors were primarily interested in features that could fulfill their information needs, i.e., survivorship care plan, access to their electronic medical record, and an overview of appointments. Health professionals considered patient reported outcomes and telemonitoring as the most useful features.
Kuijpers et al. 2016	92 breast cancer sur- vivors (mean age 49.5 years)	Four month trial of an interactive web portal	Website user satisfac- tion, patient activation score, quality of life (SF-36), and vigorous physical activity	Overview of appointments and access to the electronic medical record were most fre- quently used features and most highly valued. Average website user satisfaction was 3.8 on a 5-point scale. Patient activation scores did not change significantly. Three domains of the SF-36 (role functioning – emotional, mental health, and social functioning) and median vigorous physical activity improved significant- ly over time.
Laccetti et al. 2016	289 cancer center providers and clinic staff who performed patient portal activ- ities	Retrospective cohort	Total web portal actions and messages received	From 2009 to 2014, 289 employees per- formed 740,613 patient web portal actions and received 117,799 messages. Seventy-sev- en percent of actions were performed by nurses, 11% by ancillary staff, 6% by midlevel providers, and 5% by physicians. On average, 6.3 staff web portal actions were performed per patient-initiated message.
Groen et al. 2017	37 lung cancer pa- tients (mean age 59.6 years)	Questionnaires, a focus group, and analysis of interactive patient portal log data	Quality of life (SF-36) and patient activation score, and physical activity	The majority of responses (82%) about using the interactive patient portal were positive; 69% saw it as a valuable addition to care, and 56% perceived increased control over their health. No significant changes were observed in the outcome measures.
Alpert et al. 2018	35 cancer patients and 13 oncologists	In-depth, semi-struc- tured interviews aimed at better understanding communicative behav- iors and perceptions of a patient web portal and how it is utilized in oncology	Thematic analysis was used to examine the responses	Portals help to enhance participation during in-person consultations, increase patients’ self-advocacy, and build rapport with provid- ers. Patients’ level of comfort with reviewing information via the portal depended upon the severity of the test. Oncologists worried about patient anxiety and widening health dispar- ities, but noted that the portal can motivate them to expedite communication about test results.
Schultz & Alderfer, 2018	19 caregivers of chil- dren with cancer	One-on-one semi-struc- tured interviews	Inductive qualitative content analysis	Caregivers recognized advantages of portal use including getting results “fast,” being able to visualize trends in results, “keeping a record,” and not interfering with clinic flow. Perceived disadvantages included the results being “com- plicated” or easily misunderstood, and learning results prior to disclosure by care team.
